# Prognosis Impact of Diabetes in Elderly Women and Men with Non-ST Elevation Acute Coronary Syndrome

**DOI:** 10.3390/jcm10194403

**Published:** 2021-09-26

**Authors:** Pablo Díez-Villanueva, Jose María García-Acuña, Sergio Raposeiras-Roubin, Jose A. Barrabés, Alberto Cordero, Manuel Martínez-Sellés, Alfredo Bardají, Francisco Marín, Juan M. Ruiz-Nodar, Nuria Vicente-Ibarra, Gonzalo L. Alonso Salinas, Belén Cid-Alvárez, Emad Abu Assi, Frances Formiga, Julio Núñez, Eduardo Núñez, Albert Ariza-Solé, Juan Sanchis

**Affiliations:** 1 Servicio de Cardiología, Hospital Universitario La Princesa, 28006 Madrid, Spain; pablo_diez_villanueva@hotmail.com; 2 Servicio de Cardiología, Hospital Clínico Universitario de Santiago, CIBERCV, 15706 Santiago de Compostela, A Coruña, Spain; jgarciaacuna@gmail.com (J.M.G.-A.); belenalvarez85@hotmail.com (B.C.-A.); 3 Servicio de Cardiología, Hospital Álvaro Cunqueiro de Vigo, 36213 Vigo, Pontevedra, Spain; Sergio.Raposeiras.Roubin@sergas.es (S.R.-R.); eabuassi@yahoo.es (E.A.A.); 4 Servicio de Cardiología, Hospital Universitario Vall d’Hebron, Universidad Autónoma de Barcelona, CIBERCV, 08035 Barcelona, Spain; jabarrabes@vhebron.net; 5 Servicio de Cardiología, Hospital Clínico Universitario de San Juan, 03550 Alicante, Spain; acorderofort@gmail.com; 6 Servicio de Cardiología, Hospital Universitario Gregorio Marañón, CIBERCV, Universidad Europea, Universidad Complutense, 28007 Madrid, Spain; mmselles@secardiologia.es; 7 Servicio de Cardiología, Hospital Universitario de Tarragona Joan XXIII, IISPV, Universitat Rovira i Virgili, 43005 Tarragona, Spain; abardaji@comt.es; 8 Servicio de Cardiología, Hospital Clínico Universitario Virgen de la Arrixaca, IMIB-Arrixaca, CIBERCV, 30120 Murcia, Spain; fcomarino@hotmail.com; 9 Servicio de Cardiología, Hospital General Universitario de Alicante, 03010 Alicante, Spain; ruiz_jmi@gva.es; 10 Cardiology Department, Hospital General de Elche, 03203 Alicante, Spain; nuria.v.ibarra@gmail.com; 11 Servicio de Cardiología, Hospital Universitario Ramón y Cajal de Madrid, CIBERCV, 28034 Madrid, Spain; gonzalol.alonso@gmail.com; 12 Servicio de Medicina Interna, Hospital Bellvitge, L’Hospitalet de Llobregat, 08097 Barcelona, Spain; fformiga@bellvitgehospital.cat; 13 Servicio de Cardiología, Hospital Clínico Universitario de Valencia, INCLIVA, Universidad de Valencia, CIBERCV, 46014 Valencia, Spain; yulnunez@gmail.com (J.N.); enunezb@gmail.com (E.N.); 14 Servicio de Cardiología, Hospital Bellvitge, L’Hospitalet de Llobregat, 08097 Barcelona, Spain; aariza@bellvitgehospital.cat

**Keywords:** elderly, non-ST-segment elevation acute coronary syndromes, women, diabetes mellitus

## Abstract

Few studies have addressed to date the interaction between sex and diabetes mellitus (DM) in the prognosis of elderly patients with non-ST-segment elevation acute coronary syndrome (NSTEACS). Our aim was to address the role of DM in the prognosis of non-selected elderly patients with NSTEACS according to sex. A retrospective analysis from 11 Spanish NSTEACS registries was conducted, including patients aged ≥70 years. The primary end point was one-year all-cause mortality. A total of 7211 patients were included, 2,770 (38.4%) were women, and 39.9% had DM. Compared with the men, the women were older (79.95 ± 5.75 vs. 78.45 ± 5.43 years, *p* < 0.001) and more often had a history of hypertension (77% vs. 83.1%, *p* < 0.01). Anemia and chronic kidney disease were both more common in women. On the other hand, they less frequently had a prior history of arteriosclerotic cardiovascular disease or comorbidities such as peripheral artery disease and chronic pulmonary disease. Women showed a worse clinical profile on admission, though an invasive approach and in-hospital revascularization were both more often performed in men (*p* < 0.001). At a one-year follow-up, 1090 patients (15%) had died, without a difference between sexes. Male sex was an independent predictor of mortality (HR = 1.15, 95% CI 1.01 to 1.32, *p* = 0.035), and there was a significant interaction between sex and DM (*p* = 0.002). DM was strongly associated with mortality in women (HR: 1.45, 95% CI = 1.18–1.78; *p* < 0.001), but not in men (HR: 0.98, 95% CI = 0.84–1.14; *p* = 0.787). In conclusion, DM is associated with mortality in older women with NSTEACS, but not in men.

## 1. Introduction

Non-ST-segment elevation acute coronary syndrome (NSTEACS) constitutes one of the leading causes of hospital admissions and mortality in the elderly [1,2,3,4,5]. A great number of patients hospitalized for ACS are over 70 years of age, and women account for up to 30–40% of them [6,7]. Previous studies show women presenting with an acute coronary event are usually older, with a worse baseline clinical situation than men [8,9,10]. Such issues are associated with a worse prognosis [6,11,12], though women less often receive optimal medical therapies or an invasive approach [8,13]. However, different results have been reported in prior studies addressing the impact of sex on total and cardiovascular mortality in elderly patients with NSTEACS [8,14,15,16,17].

On the other hand, diabetes mellitus (DM) is often present in patients with acute coronary syndromes, associating a long-term excess risk of mortality [17,18,19]. Few studies have addressed to date the interaction between sex and DM in the prognosis of elderly patients with NSTEACS, and they have had contradictory results [20,21]. The present study comprised a pooled analysis of individual patient data that included elderly patients from 11 Spanish NSTEACS registries [22]. We aimed to study the prognostic influence of DM according to sex on 1-year mortality.

## 2. Material and Methods

### 2.1. Study Population

This is an analysis of a retrospective study comprising 11 cohorts from Spanish NSTEACS registries. Registry investigators provided individual patient data to form a pooled patient database. Details of the cohorts are reported elsewhere [22]. Briefly, the study included patients aged 70 years or older with NSTEACS. Patients were managed according to standard practice in each hospital. Decisions on medical therapy and regarding invasive management during the index hospitalization were all left at the discretion of the attending physicians.

### 2.2. End Point

The main end point of the study was all-cause mortality at a one-year follow-up. Information on mortality was collected from the hospital files or the regional mortality registry.

### 2.3. Statistical Analysis

Continuous variables were expressed by mean ± one standard deviation, while categorical variables as absolute values (percentages). Baseline characteristics according to sex were compared by independent sample *t*-test, or chi-squared tests, as appropriate. A Cox regression model for the one-year mortality was initially built with variables chosen based on previous knowledge and biological plausibility. Hospital centers were included as strata to allow each center to have its own baseline hazard. From this initial set of covariates, a more parsimonious model was developed by backward elimination while simultaneously testing each continuous variable for departure from linearity (multivariable fractional polynomial procedure). If indicated, variables were transformed with the appropriate fractional polynomials. The final model for mortality included (all main effects) age (years), sex, hyperlipidemia, prior history of acute myocardial infarction, prior history of admission for heart failure, prior history of admission for stroke, prior history of admission for chronic obstructive pulmonary disease (COPD), anemia (WHO class), admission heart rate (bpm), systolic blood pressure (mmHg), left ventricular ejection fraction (LVEF, %), estimated glomerular filtration rate (eGFR, in mL/min/1.73 m^2^), Killip class ≥ II, ST-segment deviation, bundle branch block and Cath and PCI procedures performed at index admission. Risk estimates were expressed as hazard ratios (HRs) with 95% confidence intervals (CIs). Proportionality assumption for the hazard function over time was tested by means of Schoenfeld residuals. The discriminative ability of the multivariate model was evaluated with Harrell’s C-statistics. Based on prior knowledge that diabetes has shown a differential prognostic effect on mortality and other outcomes based on sex, we decided to explore such an interaction. Kaplan–Meier curves were estimated, stratifying the patient population according to sex and DM.

Stata 15.1 (Stata Statistical Software, Release 15 (2017); StataCorp LP, College Station, Texas, TX, USA), was used for the main analyses. 

### 2.4. Ethics

The study complied with the Declaration of Helsinki, and the ethics committee of each participating hospital approved the study protocol.

## 3. Results

### Patient Population

A total of 7211 patients were included in the study, including 2768 (38.4%) women. Baseline characteristics according to sex are shown in Table 1. Compared with men, women were significantly older (79.95 ± 5.75 vs. 78.45 ± 5.43 years, *p* = 0.0001) and more often had a history of previous hypertension, with no differences regarding DM or dyslipidemia. On the other hand, they less frequently had a prior history of arteriosclerotic cardiovascular disease (prior myocardial infarction, and history of percutaneous coronary intervention or coronary artery bypass graft; all *p* = 0.0001) and comorbidities such as peripheral artery disease or chronic pulmonary disease. Still, anemia and chronic kidney disease were both more common in women.

On admission, women showed a worse clinical profile, with a higher heart rate and worse Killip class, though they had otherwise better left ventricular ejection fraction. Regarding invasive approach and in-hospital revascularization, they were both more often performed in men (*p* = 0.0001). At discharge, men more frequently received antiplatelet therapy and statins when compared with women.

1090 patients (15%) had died at the one-year follow-up, 702 (15.8%) men and 388 (14.6%) women, with no differences by sex in the univariate analysis. Table 2 shows the predictors of mortality at the one-year follow-up in the multivariable analysis. After adjusting for baseline differences, male sex was predictive of mortality (HR = 1.15, 95% CI 1.01 to 1.32, *p* = 0.035).

**Figure 1:**
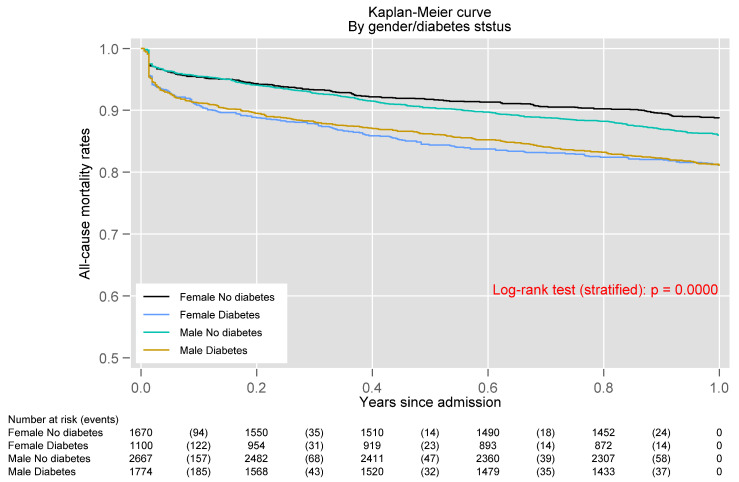
Kaplan–Meier curves for one-year mortality after stratifying patient population according to sex and Diabetes mellitus.

**Figure 2:**
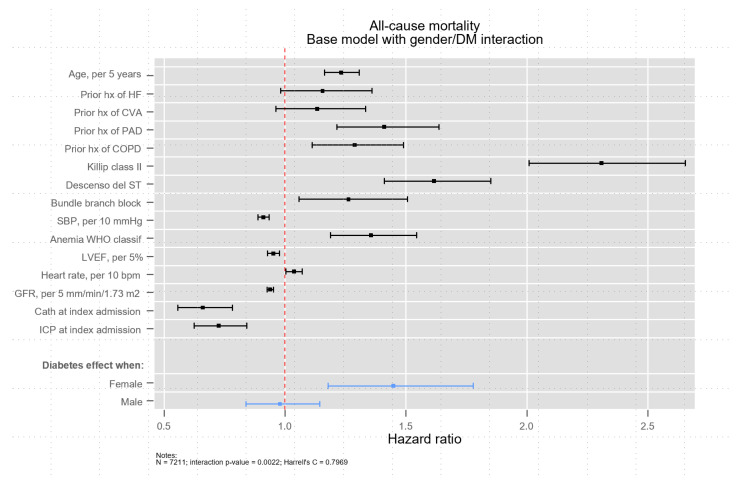
Independent predictors of all-cause mortality.

As shown in the multivariable analysis for one-year mortality, there was a significant interaction between female sex and DM for one-year mortality (*p* = 0.002). Figure 1 shows the Kaplan–Meier curves after stratifying patient population according to sex and DM. Mortality was higher in diabetics, both men and women, but the effect was greater in women (*p* = 0.0001; stratified log-rank test). Indeed, after adjusting for other predictive covariates, DM was strongly associated with mortality in women (HR: 1.45, 95% CI = 1.18–1.78; *p* < 0.001), while it was not in men (HR: 0.98, 95% CI = 0.84–1.14; *p* = 0.787) (Figure 2).

On the other hand, age, prior stroke, peripheral artery disease, admission Killip class ≥ II, ST-segment deviation, left ventricular ejection fraction, admission systolic blood pressure, admission heart rate, anemia on admission, glomerular filtration rate and invasive coronary angiography were found to be independent predictors of mortality in patients with DM (Table S1, available in Supplementary Material).

## 4. Discussion

The main findings of this study are: (1) male sex was significantly associated with worse prognosis in elderly patients with NSTEACS; (2) the prognostic impact of DM in all-cause mortality was only seen in women.

Women constitute a great proportion of elderly patients with ACS. However, they are often underrepresented in large clinical trials [23,24,25]. In accordance with previous studies, women in our series were significantly older, and age *per se* has been shown to be associated with a worse prognosis [8,9,10]. A prior history of hypertension, together with higher blood pressure on admission, were both more often found in women, and such factors were associated with better outcomes at the one-year follow-up [17]. Instead, smoking and a history of prior coronary or peripheral artery disease were all more frequent in men and both issues related to worse clinical results [14,17,26]. Additionally, women showed a worse clinical profile on admission, with higher heart rate and Killip class (both issues significantly related to one-year mortality, the latter with the higher hazard ratio in the multivariable analysis) [8]. Conditions such as anemia and chronic kidney disease were both more frequent in women, findings similar to those previously reported, and they are also associated with a worse prognosis [8,15]. On the other hand, left ventricular ejection fraction was significantly lower in men. Despite the above, however, an invasive approach and in-hospital revascularization were both more often performed in men, as previously reported [6,8,15,26,27]. Moreover, revascularization has been associated with lower short-term mortality in elderly patients with NSTEACS [28].

In our study, several conditions were found to be independently associated with a worse prognosis in the multivariable analysis, as described in previous studies [8]. Male sex was associated with higher one-year mortality but, remarkably, an interaction was found between female sex and DM, in such a way that the presence of DM conferred a much worse prognosis in elderly women with NSTEAC when compared with men with DM. Different studies have demonstrated a long-term excess risk of mortality associated with this comorbidity following NSTEACS, independent of other factors such as other cardiovascular risk factors or therapies [17,18,19]. From a pathophysiological point of view, differences regarding cardiovascular outcomes have been described between men and women with DM, since microvascular complications seem to be more frequent in men and macrovascular complications are the leading cause of both morbidity and mortality in women [29,30]. In addition, differences in management and treatment of cardiovascular risk factors have been suggested in patients with DM, substantially worse in women, thus associating with a more adverse cardiovascular profile and further events [29]. In this regard, women in our study less frequently received antiplatelet therapy and statins at discharge, similar to findings reported in previous studies [15,31]. Additionally, consistent with previous findings [28], an invasive approach, especially when performed early after admission, was significantly associated with lower mortality at the one-year follow-up in our series. Prior studies addressing the impact of revascularization in elderly patients with NSTEACS have demonstrated an obvious benefit in this clinical setting, as it is associated with lower in-hospital mortality [8,26,27,32] regardless of the presence of DM [32]. However, this strategy was less often performed in women in our series, something already reported in previous studies. In the study by Vogel et al. [8], DM was also significantly associated with conservative management, but only in men. These features, altogether, strengthen previous studies demonstrating women with DM show a more adverse cardiovascular risk profile which, combined with receiving standard treatment less often, entail worse prognoses [33].

Few studies have addressed to date the interaction between sex and DM in the prognosis of patients with NSTEACS. Koek et al. included more than 20,000 patients with a first myocardial infarction (mean age much lower than that of our series, 33% women, 9.3% DM) and found no sex differences in short and long-term mortality [20]. On the other hand, the study by Icks et al., using data from the population-based MONICA/KORA Myocardial Infarction Registry (the study population was limited to patients aged 25–74 years old), included 16,478 patients with a first fatal or non-fatal myocardial infarction (with and without ST-segment elevation) between 1985 and 2009 (mean age 62 years, 71% male, 29% with DM), and they showed a strong relationship between DM and short and long-term mortality after a first ACS, relatively higher in women, somehow in line with our findings [21]. Of note, patients included in that study were much younger than those of our series (mean age 79 years, 38.4% women) and had no documented previous coronary artery disease. Additionally, there are some temporary differences in the definition of myocardial infarction, according to different guidelines, and, importantly, data regarding revascularization are not shown. These issues, together with a much more robust multivariable analysis, make our study more solid and our results more generalizable. On the other hand, in another scenario such as acute heart failure with preserved left ventricular ejection fraction, diabetes conferred a higher risk of mortality in women [34]. Recently, Alkhouli et al. found adjusted odds of death for women vs. men varied by age in this setting, with a more pronounced negative impact of female sex on most outcomes in young and middle-aged women [35], thus reinforcing our findings.

Our study has some limitations. First, this is a retrospective study; thus, selection bias cannot be ruled out, as in all observational registries. Second, since our study comprises a pooled patient database from 11 different cohorts, issues such as type or duration of diabetes, or its complications, were not available in all of them and were not included in the analysis. Moreover, for this reason, information about treatment at discharge other than antiplatelets and statins is not available in our study. Finally, conditions with prognostic impact in elderly women with NSTEACS, such as frailty and other geriatric syndromes [36,37,38], were not included in our study. In spite of these limitations, and to the best of our knowledge, this is the first study addressing the impact of the interaction between sex and DM in the prognosis of elderly patients with NSTEACS. Our study also retrieved interesting and novel data about the prognosis of DM according to sex in this clinical scenario. Improving clinical management of these patients may contribute to improving both their quality of life and outcomes, given the global prevalence of diabetes and vascular complications. Further studies are needed to confirm these findings.

## 5. Conclusions

In conclusion, DM is associated with mortality in elderly women with NSTEACS, but not in men.

## Figures and Tables

**Table 1:** Baseline demographic and clinical characteristics according to sex.

	Total (*n* = 7211)	Male *n* = 4443 (61.6%)	Female *n* = 2768 (38.4%)	*p*
Baseline characteristics				
Age (years)	79.03 ± 5.6	78.45 ± 5.43	79.95 ± 5.75	<0.001
Diabetes	2874 (39.9)	1774 (39.9)	1100 (39.7)	0.863
Hypertension	5723 (79.4)	3420 (77)	2303 (83.1)	<0.001
Dyslipidemia	4262 (59.1)	2641 (59.5)	1621 (58.5)	0.431
Smoking habit	621 (8.6%)	536 (12,1)	85 (3.1)	<0.001
Prior myocardial infarction	1682 (23.3)	1186 (26.7)	496 (17.9)	<0.001
Prior percutaneous coronary intervention	1334 (18.5)	943 (21.2)	391 (14.1)	<0.001
Prior coronary artery bypass graft	573 (7.9)	461 (10.4)	112 (4)	<0.001
Prior admission for heart failure	641 (8.9)	383 (8.6)	258 (9.3)	0.328
Admission systolic blood pressure, mmHg	141 ± 25	140 ± 24	143 ± 25	<0.001
Admission heart rate, bpm	79 ± 19	77 ± 18	80 ± 18	<0.001
Admission Killip class ≥ II	1889 (26.2)	1118 (25.2)	771 (27.8)	0.013
ST segment deviation	2638 (37)	1633 (36.8)	1005 (36.3)	0.688
Left bundle branch block or permanent pacemaker	1147 (16)	774 (17.4)	373 (13.5)	<0.001
Troponin elevation	5319 (74)	3214 (72.4)	2105 (76)	0.001
Left ventricular ejection fraction, %	54 ± 11	53.7 ± 11	55.7 ± 10	<0.001
Comorbidities				
Prior stroke	831 (11.5)	524 (11.8)	307 (11.1)	0.363
Peripheral arterial disease	1006 (14)	791 (17.8)	215 (7.8)	<0.001
Chronic pulmonary disease	1161 (16.1)	885 (19.9)	276 (10)	<0.001
Hemoglobin on admission, gr/dl	12.9 ± 1.8	13.3 ± 1.9	12.6 ± 1.6	<0.001
Glomerular filtration rate (mL/min)	66 ± 26	69 ± 27	61 ± 24	<0.001
Invasive management				
Invasive coronary angiography	6032 (84)	3844 (86.6)	2188 (79)	<0.001
Percutaneous coronary intervention	3867 (53.6)	2552 (57.5)	1315 (47.5)	<0.001
Coronary artery bypass graft	491 (6.8)	375 (8.4)	116 (4.2)	<0.001
In-hospital revascularization	4339 (60.2)	2914 (65.6)	1425 (51.4)	<0.001
Treatment at discharge				
Aspirin	6194 (90.6)	3882 (92.2)	2312 (88.1)	<0.001
Clopidogrel	4341 (63.5)	2764 (65.6)	1577 (60.1)	<0.001
Ticagrelor	327 (4.8)	238 (5.7)	89 (3.4)	<0.001
Prasugrel	38 (0.6)	31 (0.7)	7 (0.3)	0.011
Statins	5938 (86.9)	3709 (88.1)	2229 (84.9)	<0.001

**Table 2:** Predictors of mortality at one-year follow-up.

Variable	Univariate Analysis		Multivariate Analysis (without Interaction)	
	Hazard Ratio (95% CI)	*p* Value	Hazard Ratio (95% CI)	*p* Value
Age (per year)	1.07 (1.06–1.09)	<0.001	1.04 (1.03–1.05)	0.0001
Male sex	1.12 (0.99–1.27)	0.075	1.15 (1.01–1.32)	0.035
Diabetes mellitus	1.53 (1.36–1.72)	<0.001	1.12 (0.99–1.27)	0.071
Prior admission for heart failure	2.86 (2.47–3.32)	<0.001	1.16 (0.98–1.36)	0.068
Prior stroke	1.67 (1.43–1.96)	<0.001	1.13 (0.96–1.34)	0.121
Peripheral arterial disease	2.07 (1.80–2.38)	<0.001	1.39 (1.20–1.61)	0.0001
Chronic pulmonary disease	1.67 (1.46–1.92)	<0.001	1.28 (1.11–1.49)	0.001
Admission Killip class ≥ II	4.41 (3.91–4.97)	<0.001	2.29 (1.99–2.64)	0.0001
ST segment deviation	1.95 (1.72–2.19)	<0.001	1.61 (1.41–1.85)	0.0001
Left bundle branch block or permanent pacemaker	1.33 (1.15–1.55)	<0.001	1.26 (1.05–1.51)	0.01
Left ventricular ejection fraction, %	0.96 (0.95–0.97)	<0.001	0.989 (0.985–0.995)	0.0001
Admission systolic blood pressure, mmHg	0.985 (0.982–0.988)	<0.001	0.991 (0.988–0.993)	0.0001
Admission heart rate, bpm	1.109 (1.017–1.022)	<0.001	1.003 (1.0002–1.0066)	0.034
Anemia on admission	2.45 (2.17–2.76)	<0.001	1.35 (1.19–1.55)	0.0001
Glomerular filtration rate (mL/min)	0.976 (0.974–0.979)	<0.001	0.987 (0.984–0.990)	0.0001
Invasive coronary angiography	0.36 (0.32–0.41)	<0.001	0.66 (0.56–0.78)	0.0001
Percutaneous coronary intervention	0.52 (0.46–0.59)	<0.001	0.73 (0.63–0.84)	0.0001

## Data Availability

The data presented in this study are available on request from the corresponding author. The data are not publicly available due to privacy and ethical reasons.

## Supplementary Materials

The following are available online at https://www.mdpi.com/article/10.3390/jcm10194403/s1, Table S1: Independent predictors of mortality in patients with diabetes mellitus in multivariate analysis.
