# Supplementary material for: Prognosis Impact of Diabetes in Elderly Women and Men with Non-ST Elevation Acute Coronary Syndrome
Source: J Clin Med. 2021 Sep 26;10(19):4403. doi: 10.3390/jcm10194403 (PMC8509190; doi:10.3390/jcm10194403)
Supplement: Supplementary file 1 [file jcm-10-04403-s001.zip › jcm-1381244 supplementary updated.pdf]

Journal of
Clinical Medicine

Table S1. Independent predictors of mortality in patients with diabetes mellitus in multivariate analysis.

	Hazard ratio (95% CI)	*P* value
Age	1.03 (1.01–1.05)	<0.001
Prior stroke	1.25 (1.01–1.54)	0.037
Peripheral arterial disease	1.38 (1.14–1.67)	0.001
Admission Killip ≥ II	2.36 (1.94–2.87)	<0.001
ST segment deviation	1.43 (1.19–1.70)	0.001
Left ventricular ejection fraction, %	0.990 (0.982–0.997)	0.004
Admission systolic blood pressure, mmHg	0.990 (0.987–0.994)	0.001
Admission heart rate, bpm	1.005 (1.001–1.009)	0.028
Anaemia on admission	1.24 (1.03–1.49)	0.022
Glomerular filtration rate (ml/min)	0.986 (0.982–.990)	0.001
Invasive coronary angiography	0.55 (0.45–0.66)	0.001
